# Identification of New Dystroglycan Complexes in Skeletal Muscle

**DOI:** 10.1371/journal.pone.0073224

**Published:** 2013-08-08

**Authors:** Eric K. Johnson, Bin Li, Jung Hae Yoon, Kevin M. Flanigan, Paul T. Martin, James Ervasti, Federica Montanaro

**Affiliations:** 1 Center for Gene Therapy, the Research Institute at Nationwide Children’s Hospital, and The Ohio State University Biochemistry Program, Columbus, Ohio, United States of America; 2 Ohio State Biochemistry Program, the Ohio State University, Columbus, Ohio, United States of America; 3 Department of Biochemistry, Molecular Biology and Biophysics, University of Minnesota, Minneapolis, Minnesota, United States of America; 4 Department of Pediatrics, the Ohio State University College of Medicine, Columbus, Ohio, United States of America; Goethe University, Germany

## Abstract

The dystroglycan complex contains the transmembrane protein β-dystroglycan and its interacting extracellular mucin-like protein α-dystroglycan. In skeletal muscle fibers, the dystroglycan complex plays an important structural role by linking the cytoskeletal protein dystrophin to laminin in the extracellular matrix. Mutations that affect any of the proteins involved in this structural axis lead to myofiber degeneration and are associated with muscular dystrophies and congenital myopathies. Because loss of dystrophin in Duchenne muscular dystrophy (DMD) leads to an almost complete loss of dystroglycan complexes at the myofiber membrane, it is generally assumed that the vast majority of dystroglycan complexes within skeletal muscle fibers interact with dystrophin. The residual dystroglycan present in dystrophin-deficient muscle is thought to be preserved by utrophin, a structural homolog of dystrophin that is up-regulated in dystrophic muscles. However, we found that dystroglycan complexes are still present at the myofiber membrane in the absence of both dystrophin and utrophin. Our data show that only a minority of dystroglycan complexes associate with dystrophin in wild type muscle. Furthermore, we provide evidence for at least three separate pools of dystroglycan complexes within myofibers that differ in composition and are differentially affected by loss of dystrophin. Our findings indicate a more complex role of dystroglycan in muscle than currently recognized and may help explain differences in disease pathology and severity among myopathies linked to mutations in DAPC members.

## Introduction

The dystroglycan complex is comprised of a single-pass transmembrane protein, β-dystroglycan that anchors a highly glycosylated extracellular protein, α-dystroglycan, to the membrane [1,2]. In skeletal muscle, the dystroglycan complex is an essential component of the larger dystrophin-associated protein complex (DAPC) [3]. Within the DAPC, α-dystroglycan binds to extracellular matrix proteins including laminins while the short intracellular domain of β-dystroglycan interacts with dystrophin that in turn binds to F-actin [4–6]. Therefore in striated muscles the dystroglycan complex provides a link between the intracellular cytoskeleton and the extracellular matrix that is essential for protecting the myofiber membrane from the mechanical stress imposed by muscle contraction [1,5,7,8]. Indeed, mutations that abrogate expression of dystrophin or impair binding of α-dystroglycan to the extracellular matrix lead to usually severe forms of muscular dystrophy associated with myofiber degeneration [9–14]. These observations support to the notion that the DAPC, and in particular the dystroglycan complex within it, serves an essential structural function within the muscle fiber membrane.

It is commonly believed that the vast majority of dystroglycan at the myofiber membrane is bound to dystrophin and that its main function is to link dystrophin to the extracellular matrix. The experimental evidence stems from the observation that loss of dystrophin in mice (*mdx*) and humans (DMD) leads to a dramatic decrease in dystroglycan expression at the myofiber membrane [1,15–17]. The residual dystroglycan is believed to interact with utrophin, a homolog of dystrophin whose expression is normally restricted to the neuromuscular junction (NMJ) in adult muscles [18–20]. At this site dystroglycan plays an important role in the clustering and subsequent stabilization of acetylcholine receptors at the post-synaptic membrane [21–23]. Upon loss of dystrophin expression, utrophin is up-regulated and is highly expressed throughout the membrane of regenerating myofibers [24,25]. Additionally, in non-regenerating fibers, utrophin expression is no longer restricted to the NMJ but appears to be redistributed along the sarcolemmal membrane [26,27]. This up-regulation of utrophin expression is believed to stabilize dystroglycan complexes allowing for partial functional compensation. Indeed, loss of both dystrophin and utrophin in double knockout (*mdx/Utrn* ^*-/-*^) mice leads to a much more severe muscular dystrophy than observed in *mdx* mice [28,29]. Here we provide evidence for the first time that a large fraction of dystroglycan complexes present at the myofiber membrane do not interact with dystrophin or utrophin in wild type muscles. In addition, we found that a subset of dystroglycan complexes not directly bound to dystrophin are none-the-less destabilized in the absence of dystrophin, consistent with decreased dystroglycan expression in dystrophin-deficient muscles. These findings suggest new functions for dystroglycan in muscle, with new potential implications for muscular dystrophies.

## Results

### Only a small subset of dystroglycan complexes contain dystrophin in wild type muscles

We previously observed that antibodies to dystrophin co-immunoprecipitated less dystroglycan compared with the complete co-immunoprecipitation of dystrophin by dystroglycan antibodies [30,31]. To further investigate this unusual observation, we optimized our protocol for immunodepletion of either dystrophin or dystroglycan from non-ionic detergent lysates of quadriceps muscles. Controls for non-specific protein binding to immunoglobulins were performed using an irrelevant isotype-matched mouse monoclonal antibody on wild type protein lysates (Ctr IP). Dystrophin and β-dystroglycan were not detected in any control immunoprecipitation.

**Figure 1:**
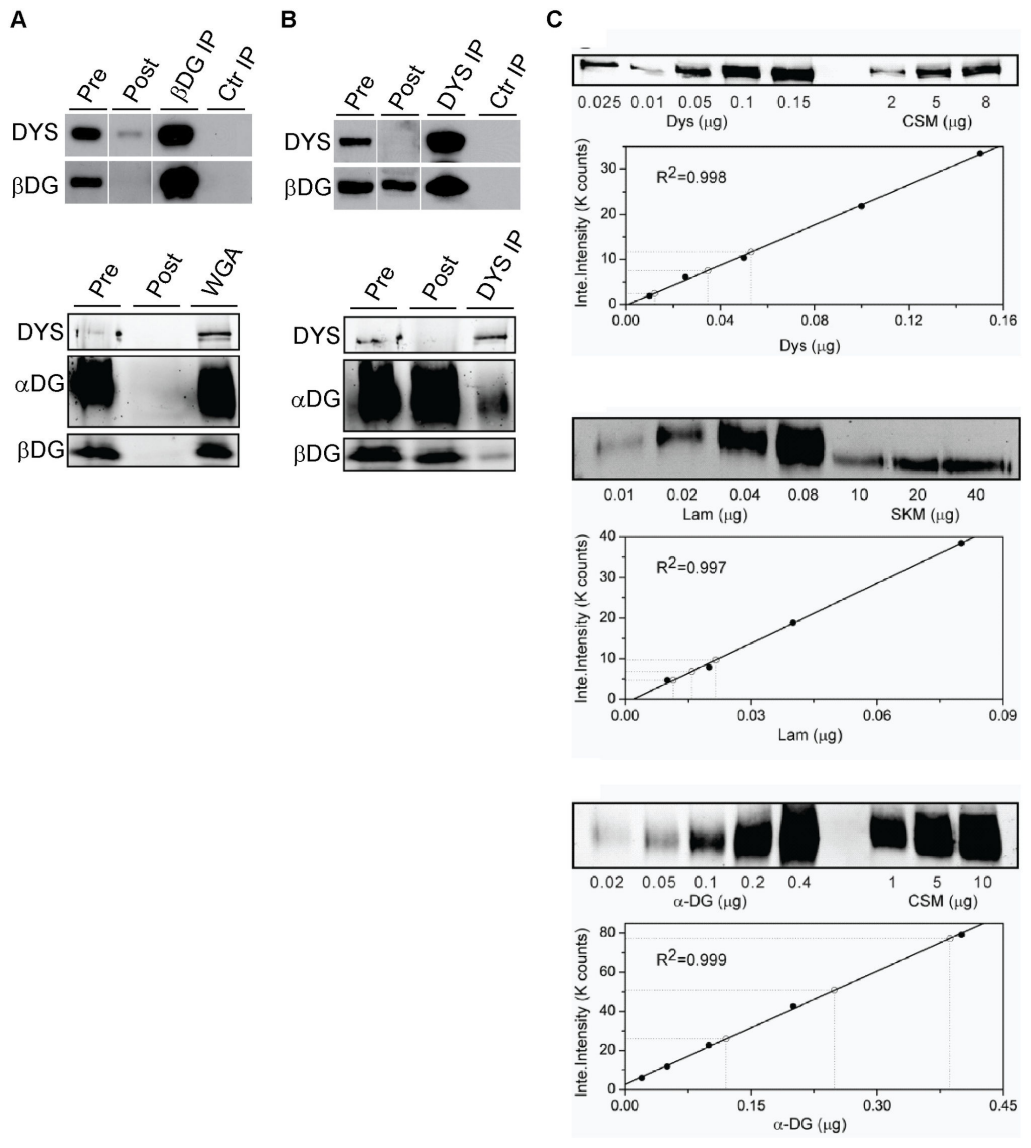
A significant pool of dystroglycan complexes are not bound to dystrophin in wild type muscle. A. Representative Western blot of β-dystroglycan immunoprecipitation (βDG IP; top) or wheat germ agglutinin pull down (WGA; bottom) of α-dystroglycan on wild type skeletal muscle lysates. Blots were probed for dystrophin (DYS), β-dystroglycan (βDG), and α-dystroglycan (αDG). Lysates before (Pre) and after (Post) were used to confirm immunodepletion of all proteins. Experiments were performed in triplicates. B. Representative Western blot of dystrophin immunoprecipitations (DYS IP) using antibodies against different domains of dystrophin (Top: MANDYS1; Bottom: DYS1). Immunodepletion of dystrophin from lysates after immunoprecipitation does not immunodeplete β-dystroglycan or α-dystroglycan. Control immunoprecipitations (Ctr IP) for both A and B used an isotype matched control antibody on wild type lysates. Experiments were performed in triplicates. C. Protein quantification of dystrophin (DYS), α-dystroglycan (αDG), and laminin (lam). Standard curves of protein band intensity were generated using purified recombinant protein, and used to calculate the amount of endogenous protein present in crude skeletal muscle membrane preparations (CSM). For laminin quantitation, only the immune signal from the common 200 kDa α1 and γ1 chains was measured in the standard curve and experimental samples.

Immunodepletion of the dystroglycan complex, either with the MANDAG2 antibody to β-dystroglycan or with wheat germ agglutinin (WGA) which binds to carbohydrate moieties on α-dystroglycan, resulted in near complete depletion of full length (427 kDa) dystrophin (Figure 1A). This indicates that the vast majority of full length dystrophin in muscle is bound to the dystroglycan complex. By contrast, when dystrophin was immunodepleted, a large amount of dystroglycan complex was detected in the post-immunoprecipitation sample (Figure 1B). Identical results were obtained with two distinct monoclonal antibodies to dystrophin directed against different regions of the protein. Therefore, only a minority of dystroglycan complexes appear to be associated with dystrophin.

Our co-immunoprecipitation results suggested that dystroglycan complex expression may markedly exceed the expression levels of dystrophin in skeletal muscle. We therefore measured the amount of dystrophin, α-dystroglycan and its extracellular binding partner laminin in mouse skeletal muscle lysates and in a sarcolemmal membrane-enriched fraction from rabbit skeletal muscle [32]. Quantitative western blot analysis against standard curves of purified proteins (Figure 1C) yielded molar dystrophin: α-dystroglycan: laminin ratios of 1:41:1 in total mouse skeletal muscle lysates (Table 1). While extraction with high salt and EDTA [32] prevented accurate measurement of laminin content in the rabbit skeletal muscle sarcolemmal vesicle-enriched membrane fraction, the molar dystrophin: α-dystroglycan ratio was measured at 1:43 (Table 1). These results suggest that the levels of dystroglycan complex in skeletal muscle is in substantial excess over dystrophin.

**Table 1:** Summary of protein concentrations for Laminin, α-dystroglycan and dystrophin in mouse skeletal muscle extracts (SKM) and rabbit crude surface membrane preparations (CSM).

	SKM	CSM
	Concentration (nmol/g SKM protein)	Concentration (nmol/g CSM protein)
Laminin	2.20 ± 0.22	N/A
α-Dystroglycan	89.72 ± 15.04	692.67 ± 57.39
Dystrophin	2.20 ± 0.07	16.11 ± 0.69
Ratio	Lam : αDG : Dys = 1 : 41 : 1	αDG : Dys = 43 : 1

The concentrations are shown as mean ± standard error with the unit of nmol per g of total mouse skeletal muscle protein (SKM) or rabbit crude surface membrane protein (CSM).

### The majority of dystroglycan in muscle is expressed by muscle cells

**Figure 2:**
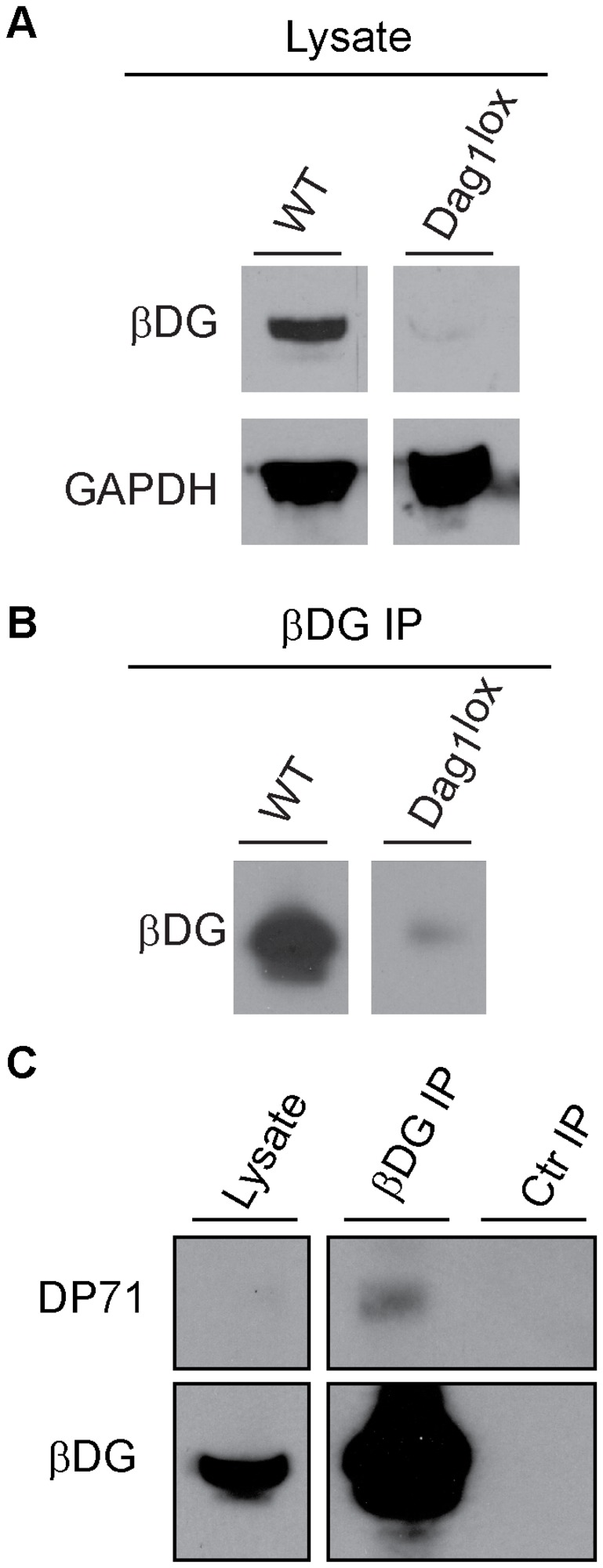
Nonmuscle cells within adult muscle do not significantly contribute to overall β-dystroglycan expression. A. Representative Western blot of β -dystroglycan (βDG) expression in total skeletal muscle lysates from wild type (WT) mice and from P3Pro-Cre;*Dag1* ^*loxP/loxP*^ mice that specifically lack dystroglycan expression in muscle cells (Dag1^loxp^). GAPDH was used as a loading control. Experiments were performed in triplicates. B. Representative Western blot of β-dystroglycan immunoprecipitations from wild type (WT) or dystroglycan deficient (Dag1^loxp^) skeletal muscle lysates probed for β-dystroglycan. Experiments were performed in triplicates. C. Representative Western blot of β-dystroglycan immunoprecipitations from wild type muscle for the presence of Dp71. No proteins were detected in non-specific antibody control immunoprecipitations (Ctr IP). Experiments were performed in duplicates.

The dystroglycan complex is not only expressed in skeletal muscle cells but also in vasculature and nerves [33–36]. At these locations, full length dystrophin is not expressed and dystroglycan interacts with utrophin or with smaller dystrophin isoforms, including Dp71 [37]. Therefore, it is conceivable that the excess dystroglycan complex observed in dystrophin immunodepletions is derived from non-muscle cells. To test for this possibility, we took advantage of *P3Pro-Cre; Dag1* ^*lox/lox*^ mice that lack dystroglycan expression specifically in hind limb myofibers and mononuclear myogenic cells [38,39]. Western blot analysis of gastrocnemius muscle lysates revealed undetectable levels of β-dystroglycan in *P3Pro-Cre; Dag1* ^*lox/lox*^ mice compared to wild type (Figure 2A). However, β-dystroglycan could be detected in these same lysates after immunoprecipitation with the MANDAG2 antibody (Figure 2B), indicating that β-dystroglycan was still expressed in non-muscle tissues but at very low levels compared to myofibers and/or myogenic cells.

To further confirm that non-muscle tissues do not significantly contribute to the pool of dystroglycan not bound to full length dystrophin, we tested whether a significant portion of dystroglycan in wild type muscle was bound to Dp71, a short dystrophin form not expressed in myofibers but present in non-muscle cells and in muscle precursor cells [37]. Dp71 was undetectable in total protein lysates from wild type muscle, but was present in very low amounts in β-dystroglycan immunoprecipitations (Figure 2C).

Together, these results suggest that a large fraction of dystroglycan complex not bound to dystrophin is present within muscle cells (myofibers and/or muscle precursors) with only a negligible contribution from non-muscle cells.

### Dystroglycan is present at the membrane of myofibers and at the neuromuscular junction in mdx and mdx/Utrn^-/-^ mice

**Figure 3:**
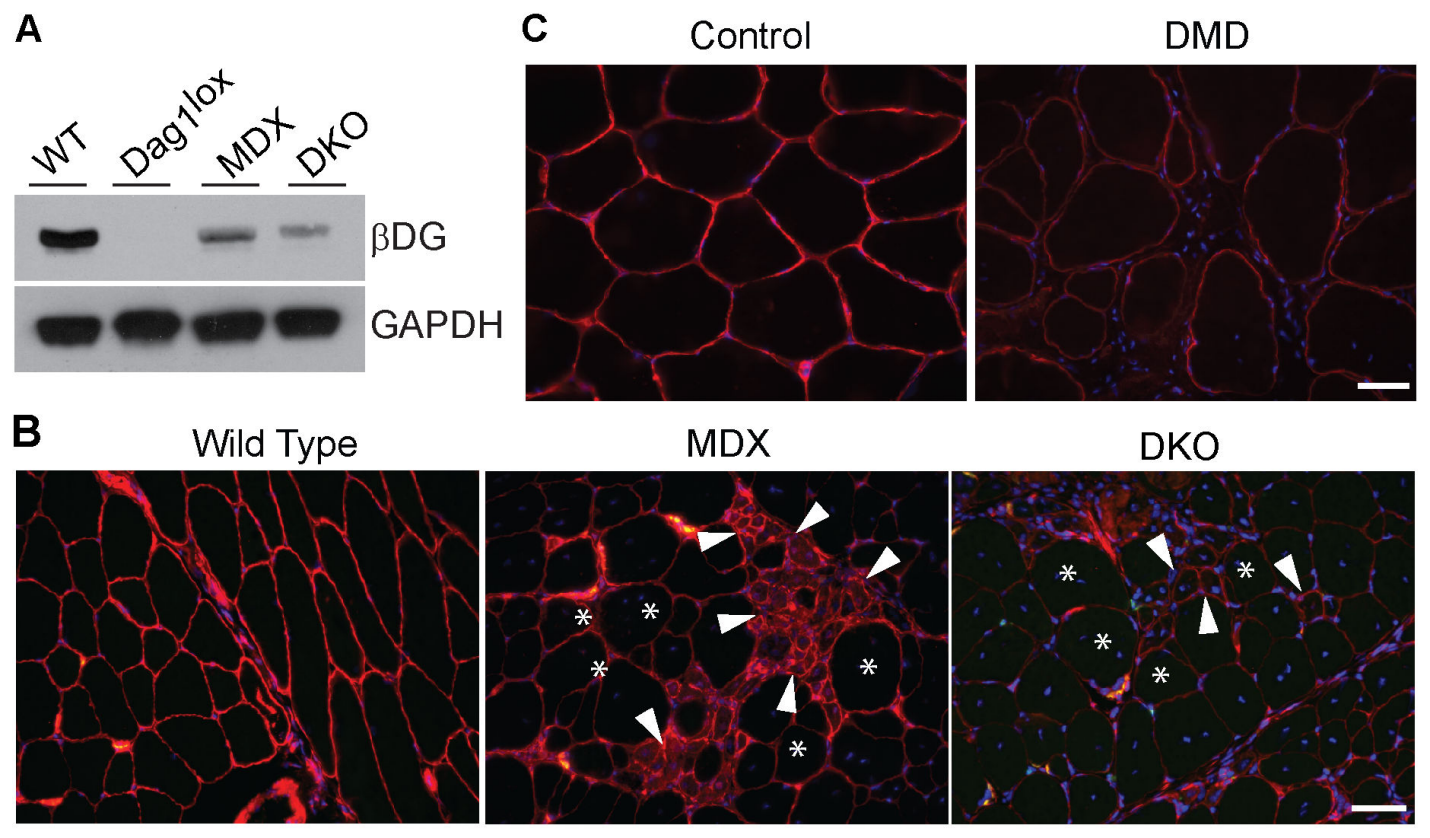
β-dystroglycan is present in myofibers of *mdx* and *mdx/Utrn^-/-^* mouse muscles and human DMD muscle. A. Representative Western blot showing β-dystroglycan (βDG) expression in total skeletal muscle lysates from wild type (WT), dystroglycan deficient (Dag1^loxp^), *mdx* and *mdx/Utrn* ^*-/-*^ (DKO) mice. GAPDH was used as a loading control. Experiments were performed in triplicates. B. Immunolabeling of wild type, *mdx* and *mdx/Utrn* ^*-/-*^ (DKO) skeletal muscle sections for β-dystroglycan (red), NMJs (green) and nuclei (blue). Arrowheads and asterisks denote non-regenerating and regenerating fibers, respectively. Micrographs were taken at the same exposure to highlight differences in staining intensity between genotypes. β-dystroglycan staining was continuous at the membrane of all muscle fibers for all genotypes (see Figure S1). C. Immunolabeling of tissue sections from control and Duchenne muscular dystrophy (DMD) biopsy samples for β-dystroglycan (red) and nuclei (blue). Scale bars: 50 µm.

If dystroglycan complexes independent of dystrophin exist within myofibers, then these complexes should be preserved in *mdx* mice that lack full length muscle dystrophin. To control for the compensatory up-regulation of utrophin in *mdx* mice, we also analyzed *mdx/Utrn* ^*-/-*^ mice, that lack both dystrophin and utrophin. By Western blot analysis of total muscle lysates, we confirmed that β-dystroglycan expression is decreased in *mdx* mice with a further visible decrease in *mdx/Utrn* ^*-/-*^ mice (Figure 3A). Interestingly, β-dystroglycan is clearly detected in both *mdx* and *mdx/Utrn* ^*-/-*^ muscle lysates in contrast to *P3Pro-Cre; Dag1* ^*lox/lox*^ mice. This observation agrees with the notion that the residual dystroglycan in *mdx* and *mdx/Utrn* ^*-/-*^ mice is expressed by muscle cells. To localize this residual β-dystroglycan, we performed immunohistochemistry on quadriceps muscle sections from wild type, *mdx* and *mdx/Utrn* ^*-/-*^ mice (Figure 2B). β-dystroglycan was detected at the neuromuscular junction in both wild type and *mdx* mice where it is believed to interact with utrophin. Interestingly, β-dystroglycan was preserved at neuromuscular junctions of *mdx/Utrn* ^*-/-*^ mice that lack both dystrophin and utrophin. In *mdx* muscle, staining for β-dystroglycan was also present in both small regenerating fibers that express utrophin (Figure 2B, arrowhead) and in large non-regenerating fibers (Figure 2B, asterisk). The intensity of β-dystroglycan staining was similar to wild type levels in regenerating fibers but was decreased in non-regenerating fibers. Even with the lowered expression, however, staining was continuous along all myofiber membranes (Figure S1). A similar result was observed in muscle sections from control versus DMD patient biopsies, where residual β-dystroglycan immunofluorescence was observed at the membrane of all myofibers (Figure 3C), although it was fainter than in control muscle. In *mdx/Utrn* ^*-/-*^ mice, β-dystroglycan was low in all fibers, regenerating and non-regenerating, but it was present in a continuous pattern along the myofiber membrane. These results indicate that a pool of β-dystroglycan is preserved at the muscle membrane and at the neuromuscular junction in the absence of both dystrophin and utrophin.

### Core DAPC members are not major components of dystroglycan complex in the absence of dystrophin and utrophin.

**Figure 4:**
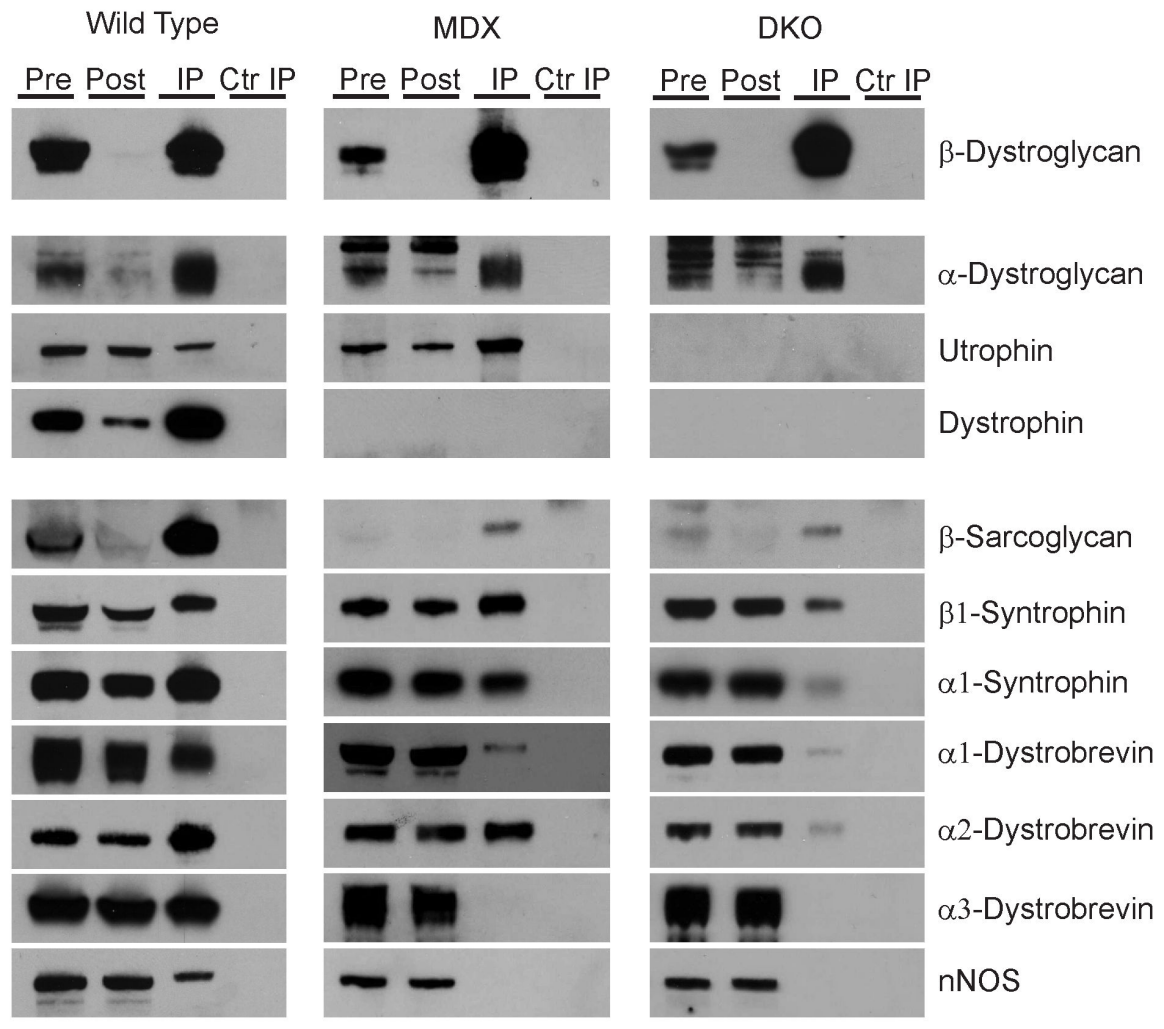
Analysis of DAPC members that associate with β-dystroglycan in wild type, *mdx* and *mdx/Utrn^-/-^* mouse muscles. Representative Western blot of β-dystroglycan immunoprecipitations (IP) from wild type, *mdx* and *mdx/Utrn* ^*-/-*^ (DKO) skeletal muscle lysates. Lysates before (Pre) and after (Post) immunoprecipitation confirmed immunodepletion of β-dystroglycan. No proteins were detected in control immunoprecipitations (Ctr IP) using an isotype matched antibody control. Membranes were cut at appropriate molecular weight sizes to allow probing of the same immunoprecipitation sample for multiple DAPC proteins. nNOS: neuronal nitric oxide synthase. Experiments were performed in triplicates.

Prior immunohistochemical studies in *mdx* muscle have shown preserved expression of some DAPC proteins at the myofiber membrane [40–42], presumably due to the compensatory up-regulation of utrophin. Here we specifically asked whether any of these residual DAPC proteins physically associate with β-dystroglycan in myofibers from *mdx* and *mdx/Utrn* ^*-/-*^ muscles. We performed immunoprecipitations for β-dystroglycan under identical conditions on lysates from wild type, *mdx* and *mdx/Utrn* ^*-/-*^ quadriceps muscles that were normalized to the same protein concentration. As shown in Figure 4, large amounts of β-dystroglycan were detected in all immunoprecipitations, regardless of genotype. In addition, for all genotypes, α-dystroglycan was strongly associated with β-dystroglycan indicating that the core dystroglycan complex is intact in both *mdx* and *mdx/Utrn* ^*-/-*^ muscles. Dystrophin was the primary intracellular binding partner of β-dystroglycan in wild type muscle compared to utrophin. As expected, dystrophin was absent in β-dystroglycan immunoprecipitations from *mdx* and *mdx/Utrn* ^*-/-*^ muscles, while utrophin co-purified with β-dystroglycan in wild type and *mdx* muscle (where it is upregulated) and was absent in *mdx/Utrn* ^*-/-*^ muscles (Figure 4).

In wild type muscle, the expected complement of DAPC proteins was readily detected in β-dystroglycan immunoprecipitations. In *mdx* muscle, nNOS, β-sarcoglycan and α3-dystrobrevin were no longer associated with the dystroglycan complex in *mdx* muscle, suggesting that these interactions may not be rescued by utrophin. In addition, α1-syntrophin and α1-dystrobrevin were reduced in β-dystroglycan immunoprecipitations from *mdx* muscle. Analysis of dystroglycan immunoprecipitations from *mdx/Utrn* ^*-/-*^ muscles revealed that loss of utrophin caused reduced or absent expression of all remaining DAPC proteins, though there was marginal preservation of β-sarcoglycan, α1- and α2-dystrobrevin, and α1- and β1-syntrophin (Figure 4).

We complemented these results with immunohistochemical analysis of DAPC proteins (Table 2, Figure S2). By and large, immunostaining of DAPC proteins in *mdx* and *mdx/Utrn* ^*-/-*^ muscle mirrored the reduced expression observed in β-dystroglycan immunoprecipitations. For example, expression of α1-dystrobrevin and β-sarcoglycan was undetectable or strongly reduced in *mdx* muscle fibers. This was the case at the membrane of both regenerating and non-regenerating muscle fibers. Interestingly, the only site where both proteins were preserved at wild type levels in *mdx* muscles was at NMJs, where utrophin is strongly expressed [26,43,44]. α1-syntrophin expression in *mdx* muscle was low in regenerating fibers, where utrophin is also highly expressed, and was low to undetectable in non-regenerating fibers. β1-syntrophin, by contrast, was highly expressed in regenerating *mdx* fibers. These observations agree with our Western blot analysis (Figure 4) showing that utrophin-dystroglycan complexes in *mdx* muscle preferentially include β1-syntrophin compared to α1-syntrophin. Interestingly, loss of dystrophin expression led to the selective loss of β1-syntrophin at the NMJ (Table 2; Figure S2). Thus, the synaptic localization of β1-syntrophin may require dystrophin and may not be compensated for by utrophin.

**Table 2:** Localization of DAPC proteins in wild type, *mdx* and *mdx/*Utrn^-/-^ mouse muscles.

	wild type			*mdx*			*mdx/Utrn* ^*-/-*^		
DAPC protein	Non-regen.	Regen.	NMJ	Non-regen.	Regen.	NMJ	Non-regen.	Regen.	NMJ
β-sarcoglycan	+++	N/A	+++	+	+	+++	+	+	-
α1-syntrophin	+++	N/A	+++	+/-	++	+++	-	++	+++*
α1-syntrophin	+++	N/A	+++	-	+++	-	-	++	-
α1-dystrobrevin	+	N/A	+++	-	-	+++	-	-	+++
α2-dystrobrevin	+++	N/A	+++	++	++	+++	-	+	+

Summary of immunohistochemical analysis on tissue sections for the indicated DAPC proteins. Representative micrographs are shown in Figure 1 and Figure S2. Non-regen: non-regenerating myofiber; Regen: Regenerating myofiber; NMJ: neuromuscular junction. +++: High expression at the membrane, similar to wild type levels; ++: slightly reduced expression; +: very low expression; +/-: very low expression in some fibers and no detectable expression in others; - : no expression detected. * α1-syntrophin expression at NMJs from *mdx/*Utrn^-/-^ mice varied and was either at levels similar to wild type or undetectable.

In agreement with our immunoprecipitations results (Figure 4), analysis of *mdx/Utrn* ^*-/-*^ muscle sections showed a more profound and general loss of immunostaining for most DAPC members, including in regenerating muscle fibers. Weak but preserved immunostaining for β-sarcoglycan, α2-dystrobrevin, and α1- and β1-syntrophins, however, was detected (Table 2; Figure S2). These same proteins were also detected at low levels in dystroglycan immunoprecipitations from *mdx/Utrn* ^*-/-*^ muscles (Figure 4). Therefore, immunostaining supported our immunoprecipitation findings, showing that loss of both dystrophin and utrophin leads to a pronounced loss of most DAPC proteins at the myofiber membrane and to their dissociation from β-dystroglycan. The latter, however, is still clearly detectable at the myofiber membrane (Figure 3) and still interacts with α-dystroglycan (Figure 4) in *mdx/Utrn* ^*-/-*^ muscle.

### Novel protein interactions of dystroglycan complexes in muscle

To gain insights into the composition of dystroglycan complexes within myofibers that do not interact with dystrophin, we applied our previously described shotgun proteomics approach [30,31] to search for proteins that co-purify with β-dystroglycan but not dystrophin. Samples from 4 independent biological replicates were analyzed on the LTQ Orbitrap and proteins identified in control immunoprecipitations were subtracted from the respective dystrophin or dystroglycan immunoprecipitation. Each experiment included two controls: an immunoprecipitation on the same lysate performed with an isotype-matched control antibody, and an immunoprecipitation with the antibody in question using muscles lacking dystrophin or dystroglycan. No known DAPC proteins were identified in any control immunoprecipitation.

Following background subtraction of both control immunoprecipitations, we focused on proteins that co-purified with β-dystroglycan in at least 3 out of 4 biological replicates, had high confidence scores and were never detected in dystrophin immunoprecipitations. Three proteins satisfied these criteria: β2-syntrophin, Cavin-1 (a.k.a. PTRF) and Cytokeratin 17 (Table 3). Among these, β2-syntrophin is already known to interact with the DAPC present at the NMJ [40,43]. This interaction is mediated by utrophin, not dystrophin, thus confirming the specificity and sensitivity of our proteomics approach [31]. In addition to these proteins, we detected several peptide matches to alpha and beta subunits of calcium channels in one β-dystroglycan immunoprecipitation (Table S1). Ion channels are not easily detected by mass spectrometry, we therefore were not surprised that these proteins were not consistently detected in all immunoprecipitations. Because the activity of calcium channels is affected in *mdx* muscle and a link has been proposed with the DAPC [45,46], we decided to test for these associations as well. Commercially available antibodies to alpha subunits did not perform well on Western blots, however, we obtained good and reproducible results with antibodies directed against the β2- and β3-regulatory subunits (Cavβ2 and Cavβ3), which are known to regulate the activity and membrane expression of several voltage gated calcium channels [47].

**Table 3:** Proteins identified by proteomics in β-dystroglycan but not dystrophin immunoprecipitations from wild type quadriceps muscles.

SWISSPROT ID	DESCRIPTION	GENE ID	Expt	SCORE	PEP.	COV.	FUNCTION
PTRF_MOUSE	Polymerase I	Ptrf	1	50	2	3.8	Membrane
	and transcript		2	207	9	16.6	repair,
	release factor		3	172	7	7.9	caveolar
	(Cavin-1)		4	45	2	2.3	formation
SNTB2_MOUSE	Beta-2-	Sntb2	1	109	6	3.1	Adaptor
	syntrophin		2	290	14	12.5	protein,
			3	78	7	3.7	signaling
			4	161	11	7.3	
K1C17_MOUSE	Keratin, type I	Krt17	1	227	20	13.6	Cytoskeletal
	cytoskeletal 17		2	151	11	6.2	protein
			3	301	10	13.6	

Expt: Experiments where the protein was detected in at least 3 out of 4 individual biological replicates; Score: Mascot protein confidence score; Pep: number of unique peptides identified; Cov: percent protein sequence coverage

We analyzed expression of the above proteins in dystrophin and β-dystroglycan immunoprecipitations from wild type muscle. In spite of high scores and lack of detection in controls samples, Cytokeratin 17 was not detected by Western blot in either dystroglycan immunoprecipitations or in total muscle lysates extracted with digitonin (data not shown). The anti-keratin 17 antibody correctly identified cytokeratin 17 in skin protein extracts, but only in the insoluble pellet fraction (data not shown), indicating that this protein is poorly extracted by digitonin. The antibody to Cavβ3 recognized a strong specific band in muscle lysates but this protein did not co-purify with β-dystroglycan or dystrophin (data not shown). We therefore did not further analyze Cytokeratin 17 or Cavβ3.

**Figure 5:**
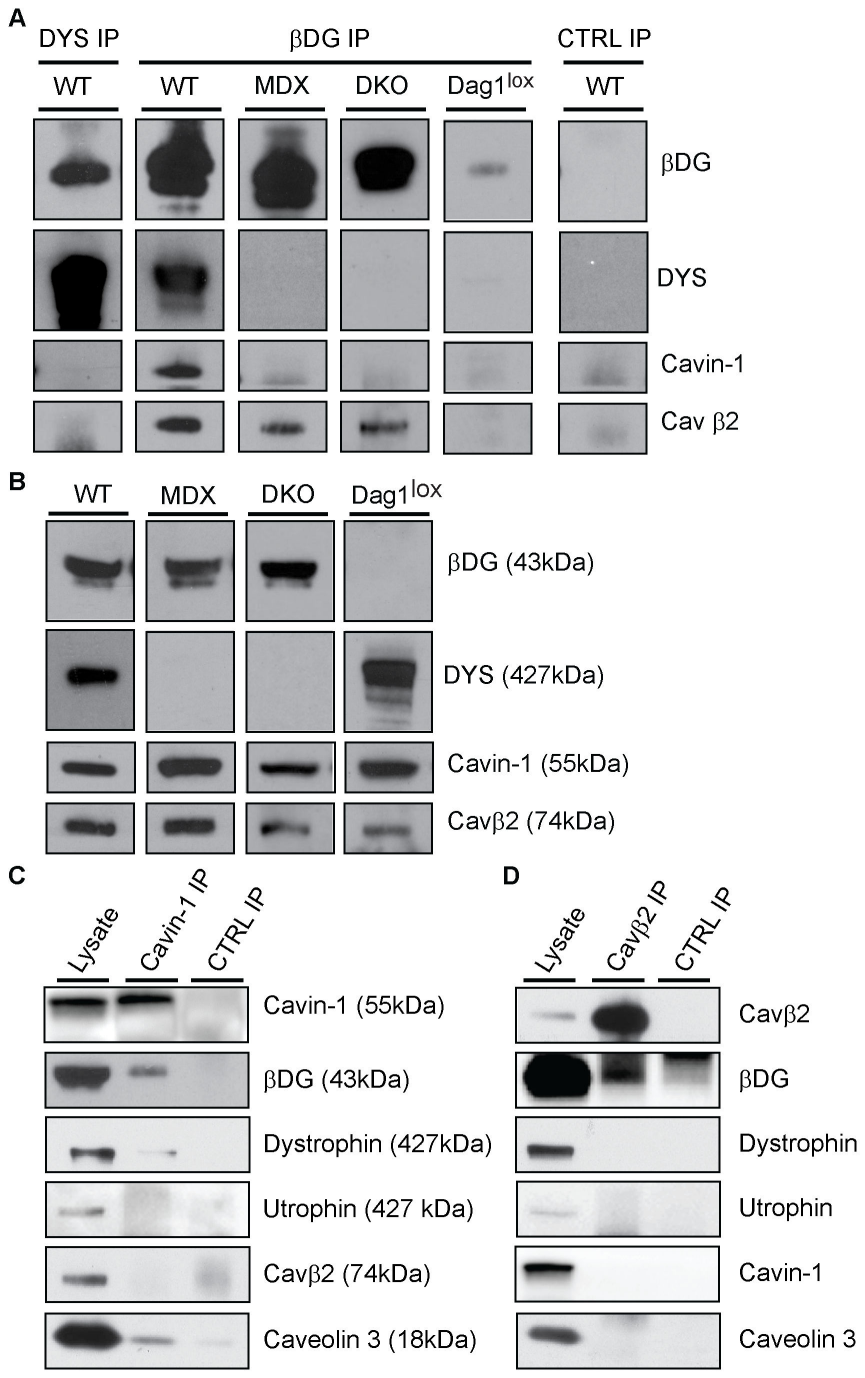
New dystroglycan-interacting proteins are differentially affected in *mdx* and *mdx/Utrn^-/-^* muscles. A. Representative Western blot of dystrophin and β-dystroglycan immunoprecipitations (DYS IP and βDG IP respectively) on skeletal muscle lysates from wild type (WT), dystroglycan deficient (Dag1^loxp^), *mdx* and *mdx/Utrn* ^*-/-*^ (DKO) mice. The membrane was cut at appropriate molecular weight sizes to allow probing of the same sample for β-dystroglycan (βDG), dystrophin (DYS), Cavin-1, and Cavβ2. No proteins were detected in control anti-β-dystroglycan immunoprecipitations on dystroglycan deficient lysates (Dag1^loxp^) or control immunoprecipitations using an isotype matched antibody on wild type lysates (Ctr IP). Experiments were performed in duplicates. B. Representative Western blot of wild type (WT), dystroglycan deficient (Dag1^loxp^), *mdx* and *mdx/Utrn* ^*-/-*^ (DKO) total skeletal muscle lysates for expression of dystroglycan (βDG), dystrophin (DYS), Cavin-1, and Cavβ2. GAPDH is shown as a loading control. Note that the more protein was loaded for the Dag1^loxp^ lysate to allow detection of Cavβ2. Experiments were performed in duplicates. C and D: Representative Western blots of reverse immunoprecipitations for Cavin-1 (C) and Cavβ2 (D) performed on wild type quadriceps muscle. Species-matched control antibodies for reverse immunoprecipitations are Rabbit-anti-Goat IgG for Cavin-1 and mouse monoclonal MW8 antibody for Cavβ2. Experiments were performed in duplicates.

By contrast, Western blot analysis confirmed that Cavin-1 and Cavβ2 did co-purify with β-dystroglycan, but not dystrophin, in wild type muscle (Figure 5A). We therefore analyzed the expression of both proteins in *mdx* and *mdx/Utrn* ^*-/-*^ muscles as well as *P3Pro-Cre; Dag1* ^*lox/lox*^ muscles where dystroglycan is selectively ablated in muscle cells [39]. Both Cavin-1 and Cavβ2 are present in total muscle lysates from *mdx, mdx/Utrn* ^*-/-*^ and *P3Pro-Cre; Dag1* ^*lox/lox*^ mice (Figure 5B). Thus, loss of dystrophin, dystrophin and utrophin, or muscle dystroglycan does not lead to complete loss of expression of Cavin-1 and Cavβ2. However Cavβ2 protein levels were lower in *mdx/Utrn* ^*-/-*^ lysates, with a further decrease in expression in *P3Pro-Cre; Dag1* ^*lox/lox*^ lysates for which more protein was loaded in the Western blot shown in Figure 5B. Analysis of β-dystroglycan immunoprecipitations revealed that while the association of Cavβ2 with β-dystroglycan was preserved in both *mdx* and *mdx/Utrn* ^*-/-*^ muscles, binding of Cavin-1 to β-dystroglycan was lost in the absence of dystrophin (Figure 5A). Interestingly, we observed a decrease in the intensity of Cavin-1 immunolabeling of mdx myofiber membranes compared to wild type (Figure S3), although total Cavin-1 protein levels appear unaffected in total *mdx* muscle lysates (Figure 5B). Neither Cavin-1 nor Cavβ2 were detected in β-dystroglycan immunoprecipitations from *P3Pro-Cre; Dag1* ^*lox/lox*^ muscles, where dystroglycan is selectively lost in myofibers and myogenic cells, and no longer co-purifies with full length dystrophin (Figure 5A). In addition, we performed reverse immunoprecipitations on wild type muscle using antibodies to Cavin-1 and Cavβ2 (Figure 5C and D). A fraction of total β-dystroglycan co-purified with Cavin-1 and Cavβ2. Utrophin did not co-purify with either Cavin-1 or Cavβ2. Dystrophin was not detected in Cavβ2 reverse immunoprecipitations, but was present in very low amount in Cavin-1 immunoprecipitations. Cavin-1 was not detected in Cavβ2 immunoprecipitations and vice versa. Finally, caveolin 3, a known binding partner of Cavin-1 co-purified with Cavin-1 but not Cavβ2, confirming the specificity of the reverse immunoprecipitations. Overall, these results indicate that 1) the β-dystroglycan/ Cavβ2 interaction is independent of both dystrophin and utrophin, 2) the β-dystroglycan/ Cavin-1 complex may have a weak and remote interaction with dystrophin but appears severely affected by the absence of dystrophin based on decreased expression of membrane-associated Cavin-1, in *mdx* muscles; and 3) the β-dystroglycan/ Cavin-1 and the β-dystroglycan/ Cavβ2 complexes are present within muscle cells and are independent of each other.

### Discussion

**Figure 6:**
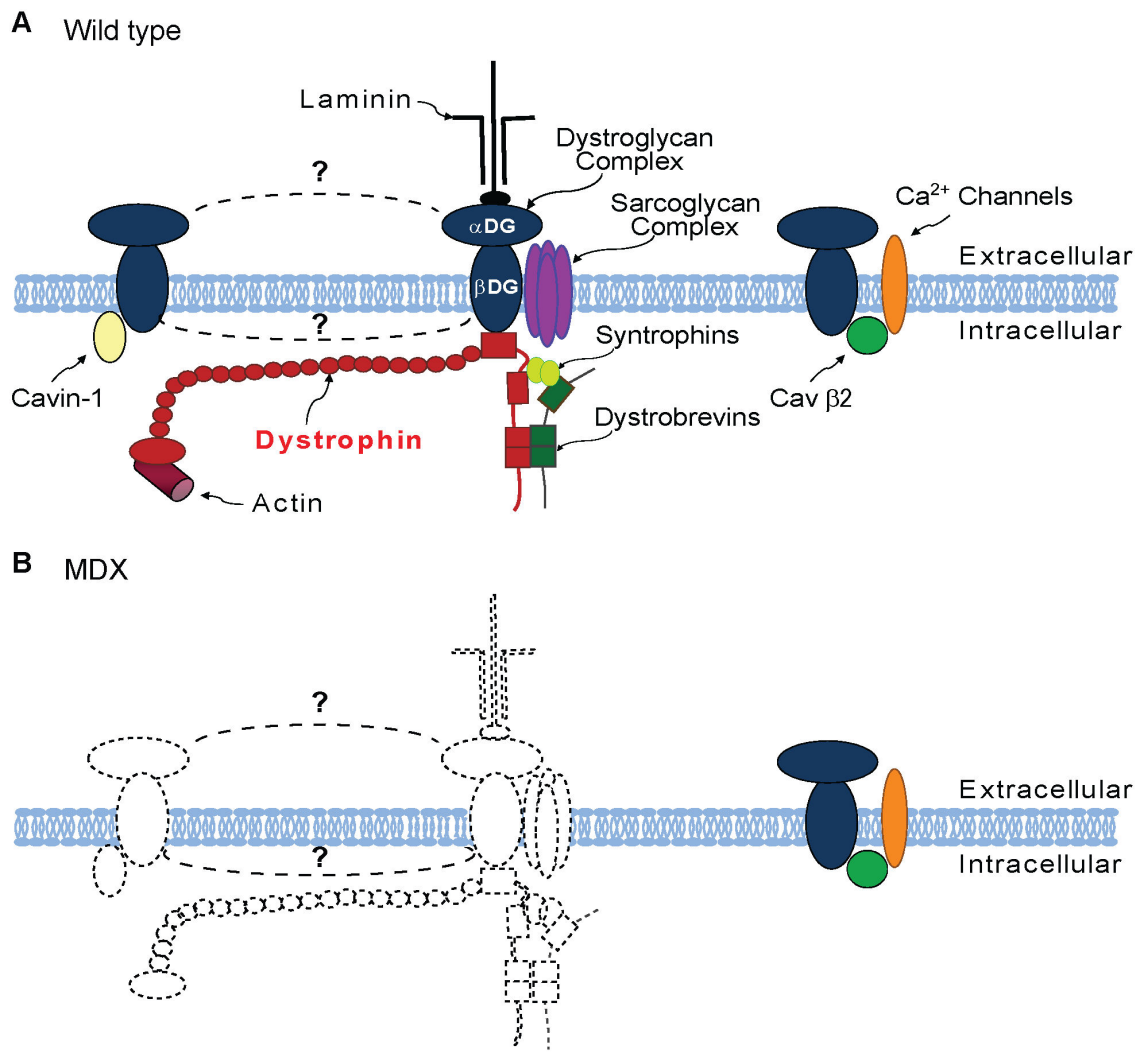
Proposed model for dystroglycan complexes in wild type and dystrophin-deficient muscle. A. Schematic representation of β-dystroglycan complexes localized at the membrane of wild type skeletal muscle fibers. B. Schematic representation of β-dystroglycan complexes at the membrane of *mdx* skeletal muscle fibers. Complexes that are perturbed in *mdx* skeletal muscles are represented by dashed lines. The mechanisms of association of the DAPC (center) and a subset of β-dystroglycan complexes (left) are not known.

Dystroglycan is a ubiquitously expressed protein that serves many functions in a diverse array of mammalian tissues. While its essential role in linking dystrophin and utrophin within the DAPC complex in muscle is well studied, little is known about other possible dystroglycan protein complexes that may subserve many of its potential functions in either non-muscle or muscle tissues. Using multiple approaches, ours is the first study to demonstrate the existence of at least three separate pools of dystroglycan complex in skeletal muscle (Figure 6). The first pool contains dystrophin as well as known core DAPC proteins such as sarcoglycans, dystrobrevins and syntrophins that co-purify with both dystrophin and dystroglycan complex [30]. Based on the measured 1:1 ratio of laminin to dystrophin in total skeletal muscle lysates, this complex is likely linked to laminin in the extracellular matrix. A second dystroglycan complex pool does not appear to strongly interact with dystrophin but is likely dependent upon dystrophin for stability at the membrane. This pool would include the β-dystroglycan/ Cavin-1 complex. Destabilization of this pool of dystroglycan complex in *mdx* muscle would account for the large decrease in dystroglycan expression and for reduced Cavin-1 membrane localization in *mdx* muscles. We currently do not understand how the stability of this pool of dystroglycan complex depends on dystrophin, and whether this is a primary or secondary consequence of loss of dystrophin. Finally, a third dystroglycan complex pool appears to function independent of both dystrophin and utrophin for its expression at the myofiber membrane and at the neuromuscular junction, and for its protein interactions. These dystroglycan complexes are preserved in dystrophic muscles and may function in part in the regulation of voltage gated calcium channels.

An unexpected finding of this study is the large abundance of dystroglycan complex that does not appear to be bound to full length dystrophin or utrophin in wild type skeletal muscle. Although we measured a 40: 1 molar ratio for the relative abundances of dystroglycan and dystrophin, our quantitation rests on several assumptions. First, the quantitation assumes that the IIH6 monoclonal antibody binds the same to α-dystroglycan from different species, although we feel we have addressed this concern by measuring the dystrophin-to-α-dystroglycan ratio in a sarcolemmal-enriched membrane fraction from rabbit skeletal muscle. Second is the assumption that purified proteins transfer with the same efficiency as proteins in more complex mixtures such as lysates. Third, our quantitation assumes that the proteins used as standards are pure or at least purified to similar extents, which is straightforward in the case of dystrophin and laminin, but problematic for α-dystroglycan because it reacts very poorly with protein stains [48]. Based on mass spectrometry analysis, contamination in our α-dystroglycan preparations was previously estimated at 5% [49], which is on par with the purities of the dystrophin and laminin-1 used as standards. While the 40:1 ratio seems high, both the protein quantitation and immunoprecipitation data independently support our conclusion that a significant amount of dystroglycan complex in skeletal muscle does not interact with dystrophin. This “free” pool of dystroglycan may not have been previously detected in prior studies due to the use of WGA to enrich for the DAPC, which does not allow distinction between dystrophin-containing and dystroglycan complexes. Evidence for the existence of such a “free” pool of dystroglycan can be found in several prior studies that used sucrose-gradient fractionations to analyze the DAPC. In these studies, dystroglycan was present in fractions that did not contain dystrophin or the sarcoglycans [50–53]. In addition, several studies have identified intracellular binding partners of β-dystroglycan that would disrupt its interaction with dystrophin. These include caveolin 3 [54], Grb2 [55], and phosphorylation of β-dystroglycan by specific kinases [56]. It has therefore been suggested that the interaction of β-dystroglycan with dystrophin may be dynamic rather than static within muscle cells, thus allowing dystroglycan to serve additional functions beyond structural integrity. Indeed, a growing number of studies in non-muscle cells have implicated dystroglycan with a variety of signaling and ion channel regulatory functions [57–66]. Our data support the notion that a significant number of dystroglycan complexes are available within muscle fibers to perform a variety of functions beyond ensuring structural integrity via dystrophin. What these functions are remains to be determined and will be the focus of future studies.

Importantly, our results further indicate that the “free” pool of dystroglycan is unlikely to be stabilized at the muscle membrane by utrophin or shorter dystrophin forms such as Dp71. Alternative proteins that may anchor these dystroglycan complexes include ankyrins and plectins that can bind directly to β-dystroglycan [67,68]. We have previously detected ankyrin in dystroglycan immunoprecipitations from *mdx* muscles [31] and association of dystroglycan with plectins can be modulated by tyrosine phosphorylation of the β-dystroglycan intracellular domain leading to amelioration of the *mdx* phenotype [15]. Although intracellular binding partners of dystroglycan that are distinct from dystrophin and utrophin likely exist in muscle, our data suggest that these interactions may be affected by loss of dystrophin in *mdx* muscle. This is based on our immunohistochemical and biochemical data indicating strongly reduced dystroglycan expression in non-regenerating muscle fibers of *mdx* muscles, and on the observed destabilization of the dystroglycan-Cavin1 complex. One possible mechanism by which loss of dystrophin may indirectly destabilize “free” dystroglycan complexes at the myofiber membrane is through the disorganization of the microtubular and sub-membranous actin networks [27,69]. Alternatively, these “free” dystroglycan complexes may be post-translationally altered in the absence of dystrophin and remain trapped in microsomes [70]. Yet a third possibility is that “free” and dystrophin-associated dystroglycan complexes are linked through some extracellular pathway, possibly involving interaction via multivalent extracellular matrix ligands that may include laminin [71,72] or biglycan [73].

Another contribution of this study is the identification of Cavin-1 and Cavβ2 as novel binding partners of dystroglycan. Cavin-1 performs a dual function in controlling caveolar dynamics and initiation of membrane repair [74,75]. We show here that Cavin-1 is in a complex with dystroglycan that may be weakly associated with dystrophin. This is in contrast to our findings in the heart where Cavin-1 is an integral part of the dystrophin complex [30]. Although in both cardiac and skeletal muscle loss of dystrophin ultimately results in loss of Cavin-1 association with the DAPC, the biological significance of assigning Cavin-1 to a dystrophin or dystroglycan complex remains unclear. Importantly, not all Cavin-1 at the myofiber membrane appears to be bound to dystroglycan. This is supported by the observation that a pool of Cavin-1 not bound to dystroglycan is detected at the membrane of *mdx* muscle fibers. In addition, dystroglycan immunodepletions do not deplete Cavin-1 (data not shown). Furthermore, dystrophin-deficient muscles show normal membrane repair [76] suggesting that the residual Cavin-1 at the membrane is functional and may therefore represent a Cavin-1 pool that does not interact with dystroglycan in wild type muscle. Interestingly, both mouse and human dystrophin-deficient muscles show increased numbers and a general disorganization of caveolae [77,78]. It is therefore conceivable that the interaction of dystroglycan with Cavin-1 in skeletal muscle may be involved in the regulation of caveolae. Of note, dystroglycan has already been shown to play a role in caveolar distribution in smooth muscle [79]. The association of a pool of dystroglycan independent of dystrophin and utrophin with Cavβ2 suggests a function in the regulation of voltage gated calcium channels. Cavβ subunits play important roles in the regulation of membrane expression of calcium channels, their gating properties and their activation kinetics [47]. Although the interaction of Cavβ2 with dystroglycan is independent of dystrophin, studies in *mdx* skeletal muscle fibers indicate that some but not all properties of L-type calcium channels are perturbed [45]. Loss of dystrophin does not appear to affect expression levels of the channels in limb muscles [80]. Instead, disturbances in calcium channel activity appear to be linked to the resulting disorganization of the sub-membranous cytoskeleton and deregulation of PKA signaling [46]. These observations indicate that alterations in L-type calcium channels are a secondary event to loss of dystrophin, a conclusion that is compatible with our biochemical findings. It will be important to determine whether more severe impairment of calcium channel function is present in dystroglycanopathies, a group of muscular dystrophies where the function of dystroglycan is the primary molecular defect [81].

We would like to note that dystrophin and known DAPC members were the primary proteins identified by proteomic profiling of dystroglycan immunoprecipitations, indicating that they are the most abundant dystroglycan-interacting proteins in skeletal muscle. However, we have shown here that dystroglycan complexes independent of dystrophin and utrophin may contain only very small amounts of DAPC proteins. One possible explanation for this apparent discrepancy is that the “free” pool of dystroglycan complexes may be highly heterogeneous in their protein interactions and therefore do not have a dominant signature by proteomics. Indeed, Cavin-1 and Cavβ2 are not abundant proteins in dystroglycan immunoprecipitations based on Western blot analysis. In addition, the list of proteins found to associate with dystroglycan in adult muscle is growing, supporting the notion that multiple dystroglycan complexes may co-exist in muscle and may be stabilized at the membrane by cytoskeletal proteins beside dystrophin and utrophin [31,67,68,82–85]. Further enrichment of these “free” dystroglycan complexes will be key to revealing their composition and gain insights into their possible functions.

Finally, our results agree with prior studies that utrophin does not restore all dystrophin protein interactions in *mdx* muscle [27,86]. As previously reported [86], we found that utrophin complexes do not include nNOS. In addition, we found that utrophin complexes lack β-sarcoglycan, α1-dystrobrevin and α3-dystrobrevin. Furthermore, from the comparison of the immunofluorescence findings among all genotypes, three interesting observations can be drawn. First, additional proteins beside utrophin must exist in regenerating myofibers for localizing and stabilizing α- and β-dystroglycans, β-sarcoglycan, α1- and β1-syntrophins, and α2-dystrobrevin at the myofiber membrane and at the neuromuscular junction, for at least a subset of these proteins. Possible candidates are ankyrins which are not efficiently extracted by detergents [67], and plectin-1, which has been shown to bind dystroglycan and F-actin [68]. Second, the requirements for utrophin and dystrophin are different between the non-synaptic membrane and the NMJ. For example, loss of dystrophin leads to a near complete loss of β-sarcoglycan at non-synaptic membranes, however its expression at the NMJ is entirely dependent upon utrophin expression. By contrast, synaptic localization of β1-syntrophin is entirely dependent on dystrophin expression; however this syntrophin interacts with utrophin in regenerating fibers. Third, the composition of both dystrophin and utrophin complexes changes depending on the location in the myofiber (synaptic versus extra-synaptic) or the developmental status of the fiber (regenerating versus non-regenerating). This suggests that there are mechanisms that are used by myofibers to regulate the temporal and spatial inclusion or exclusion of specific proteins from dystrophin and utrophin complexes. This is in agreement with our findings in heart versus limb muscles where we found that Cavin-1, Cypher, Ahnak1 and CRYAB are specifically excluded from the skeletal muscle dystrophin complex even though these proteins are highly expressed in both cardiac and skeletal muscles [30].

Overall, our study has revealed a greater complexity of regulation, composition and possibly function of the dystroglycan complex in adult muscle than currently appreciated. The challenges ahead will be to biochemically separate the different dystroglycan complexes in order to characterize their composition by proteomics and most importantly, to identify their functions in muscle maintenance and disease. These studies are fundamental because a growing number of myopathies are linked to mutations in genes that regulate the glycosylation and therefore the function of dystroglycan in muscle [87,88]. It is currently assumed that these mutations lead to muscle disease by impairing the structural functions performed by dystroglycan within the DAPC. However, the existence of dystroglycan complexes independent of the DAPC would imply that additional disease mechanisms may be involved in the highly variable clinical phenotype of dystroglycanopathies, with important implications for treatment avenues.

### Materials and Methods

#### Human biopsy samples

Archived tissue from residual clinical muscle biopsy specimens was used for immunofluorescent analysis under protocol # 0502HSE046 approved by the institutional review board of Nationwide Children’s Hospital. Written consent was obtained. DMD diagnosis was confirmed by clinical features and by mutation analysis from genomic DNA. Muscles sampled were known for the DMD patients (quadriceps [CRM] and gastrocnemius [DA]) but not for the normal control samples. Muscle was snap-frozen in isopentane cooled to liquid nitrogen temperatures as per standard protocols [89] and was stored at -80^0^C prior to cryosectioning in 10-micron thick sections.

#### Animals

Wild type mice were maintained as a C57Bl/6 strain. Mice deficient in dystrophin (*mdx* ^*5cv*^, B6Ros.Cg-Dmd^mdx-5Cv^/J, stock 002379) were a kind gift of Dr. Louis Kunkel, Harvard University and were maintained as a C57Bl/6J strain. Mice with hind limb muscle deleted for dystroglycan (*P3Pro-Cre; Dag1* ^lox/lox^) were made by crossing P3Pro-Cre transgenic mice, which show a caudal to rostral gradient of Cre transgene expression in skeletal muscles, to *Dag1* ^loxP/loxP^ mice [38,39]. Mice were bred and cared for in a clean barrier facility and all animal care and experiments were done under protocols approved by the Institutional Animal Care and Use Committee (IACUC) at Nationwide Children’s Hospital (IACUC # AR05-00025 and AR06-00052).

#### Antibodies

Anti-dystrophin (MANDYS1), isotype-matched control (MW8), and anti-β-dystroglycan (MANDAG2) antibodies were produced in-house from hybridoma cell lines (DSHB; University of Iowa) and concentrated using the Amicon ultra-filtration cell (Millipore) to be used for immunoprecipitations. Other antibodies used are: DYS1 (Novocastra) to dystrophin, a polyclonal antibody to the C-terminus of dystrophin pre-absorbed for cross-reactivity with utrophin was used to detect Dp71 (a kind gift of Jeff Chamberlain, University of Washington), isoform specific anti- α1-, or β1-syntrophin, and anti- α1- or α2-dystrobrevin antibodies [40,41,43] (a kind gift of Stan Froehner, University of Washington); clone IIH6C4 to α-dystroglycan (Millipore); anti-nNOS (#610308) and pan anti-α-dystrobrevin (#610766, BD Bioscience); anti-β-sarcoglycan (clone 5B1, Leica Microsystems); anti-utrophin (DRP2, Leica Microsystems); anti-caveolin 3 (BD transduction Labs, clone 26); anti-cavin-1 (ab40840, Abcam) anti-Ca_v_β2 (ab93606, Abcam), anti-Ca_v_β2 (ab79264, Abcam), and anti-keratin 17 (ab109725, Abcam). For Figure 1 A, B lower immunoblots, mouse monoclonal anti-dystrophin antibody Dy4/6D3 (DYS1) and anti-β-dystroglycan antibody 43DAG1/8D5 were purchased from Novocastra. For Figure 1B lower immunoprecipitation, mouse monoclonal anti-dystrophin antibody ab14452 was purchased from Abcam Inc. For quantitative western blot in Figure 1C, rabbit polyclonal anti-laminin antibody L9393 was purchased from Sigma-Aldrich.

#### Immunoprecipitations

Immunoprecipitations were performed from quadriceps, a mixed fiber type muscle, following our previously described protocol [30,31]. The MANDYS1 antibody raised against amino acids 1431-1505 of full length dystrophin was used for all dystrophin immunoprecipitations with the exception of Figure 1B where the DYS1 antibody raised against amino acids 1181-1388, was used. Control antibodies were MW8 for MANDYS1, MANDAG2 and Cavβ2 immunoprecipitations; and purified Rabbit-anti-Goat IgG antibody (Jackson Immunochemicals) for Cavin-1 immunoprecipitations. Proteins were extracted in a buffer containing 1% digitonin and 0.05% NP-40. For immunodepletions, we titrated the amount of protein in the lysate before immunoprecipitation (Pre) against a set concentration of purified MANDAG2 or MANDSY1 antibody conjugated to protein G beads. This allowed us to identify a protein concentration of 1mg as ideal for consistent immunodepletion of β-dystroglycan or dystrophin for all genotypes studied. Proteins were eluted in Laemmli Reducing Sample Buffer for Western blot analysis or in 2% SDS or 100 mM DTT for LC-MS/MS analysis.

#### WGA pull-downs

Wild-type mouse skeletal muscle were homogenized in PBS (150mM NaCl, 8mM NaH2PO4, 42mM Na2HPO4, pH 7.5) with 0.5% Triton X-100 at 4 °C with end over end mixing for 1 h. After centrifugation at 100,000 × g for 40 min, 10µg of solubilized proteins from the supernatant were loaded on 50µl Wheat Germ Agglutinin bound agarose beads (Vector Laboratories, Inc.) or on 50µl ab14452 (anti-dystrophin antibody from Abcam Inc.) coupled agarose beads (50µg antibody coupled to 50µl agarose beads using Pierce® Mammalian Co-Immunoprecipitation Kit from Thermo Fisher Scientific Inc.) respectively and incubated with end over end mixing at 4 °C overnight. The void fractions were collected and the bead-bound proteins were eluted by incubating with LSB (3% SDS, 115mM sucrose, 65mM Tris-HCl, 0.0004% bromophenol blue, pH 6.8) at 100° C for 2 minutes. Equivalent amount of loaded protein (Load), unbound protein (WGA Void and Anti-Dys Void) and bound protein (WGA Bound and Anti-Dys Bound) were analyzed by SDS PAGE and Western Blot with mouse monoclonal anti-dystrophin antibody NCL-DYS1 (Novocastra), mouse monoclonal anti-α-dystroglycan antibody IIH6C4 (Millipore Co.) and mouse monoclonal anti-β-dystroglycan antibody NCL-b-DG (Novocastra).

#### Protein quantitation

Purified mouse laminin-111 was purchased from Sigma-Aldrich, Inc. Rabbit skeletal muscle α-dystroglycan was purified as previously described [90]. Mouse recombinant full length dystrophin was expressed and purified as previously described [91]. For total skeletal muscle lysates, wild-type mouse skeletal muscles were pulverized in liquid nitrogen and solubilized in 1:4 (m: V) SDS lysis buffer (1% SDS, 5mM EGTA, 1mM Benzamadine, 10µM Leupeptin, 0.2mM PMSF) at 100° C for 2 min followed by centrifugation at 25,000 × g for 2 min. The protein concentration of the supernatant was determined with Bio-Rad D_C_ protein assay kit. Rabbit skeletal muscle crude surface membrane was prepared as described previously [32]. Increasing amounts of purified laminin-1, α-dystroglycan or dystrophin were run side by side with various amounts of mouse skeletal muscle SDS lysates or rabbit skeletal muscle crude surface membrane vesicles on a 3-12% SDS-polyacrylamide gel, transferred to nitrocellulose membrane and incubated with anti-dystrophin antibody (DYS1, in 1:50 dilution), anti-α-dystroglycan antibody (IIH64, in 1:1000 dilution) or anti-laminin-111 antibody (in 1:1000 dilution) respectively. The immunofluorescence signal was quantitatively measured with Li-Cor Odyssey® Imager system (Li-Cor Biosciences, Lincoln, NE, USA). A concentration standard curve for each blot was generated by plotting fluorescence integrated intensity against the corresponding amount of purified protein. The amount of laminin, α-dystroglycan or dystrophin in the loaded muscle samples were calculated from the standard curve. For laminin quantitation, only the immunofluorescence signal from the common 200 kDa β1 and γ1 chains was measured in the standard curve and experimental samples.

#### LC-MS/MS

Proteomic analysis was performed on 3 dystrophin immunoprecipitations and 4 β-dystroglycan immunoprecipitations from wild type muscle. Each sample is a separate biological replicate, not a technical replicate. To identify proteins that non-specifically co-purify with dystrophin or dystroglycan and subtract them from analysis, we also performed proteomic analysis on the following controls: 2 dystrophin immunoprecipitation on control *mdx* ^*5cv*^ muscles (background specific to the dystrophin antibody), 2 dystroglycan immunoprecipitations on control *P3Pro-Cre; Dag1* ^*lox/lox*^ muscles (Background specific to the dystroglycan antibody), and 3 immunoprecipitations with the MW8 antibody to Huntingtin (general IgG control for both dystrophin and dystroglycan immunoprecipitations). Eluted proteins were chloroform/methanol precipitated, resuspended in 5X Invitrosol protein solubilizer (Invitrogen), diluted with 25 mM ammonium bicarbonate to a final volume of 1X Invitrosol. The proteins were then reduced with 10 µL DTT (5 mg/mL solution in 100 mM ammonium bicarbonate) and carbamidomethylated with 10 µL Iodoacetamide solution (15 mg/mL in 100 mM ammonium bicarbonate). Trypsin (in 50 mM ammonium bicarbonate) was added to the protein solution with an enzyme to substrate ratio of 1:25 (w/w). Samples were incubated for 2 hr. at 37 °C before quenching by acidification. Capillary-liquid chromatography-nanospray tandem mass spectrometry was performed on a Thermo Finnigan LTQ Orbitrap mass spectrometer (Thermo, Fisher Scientific, San Jose CA) equipped with a microspray source (Michrom Bioresources Inc, Auburn, CA) operated in positive ion mode. Samples were loaded onto a precolumn Cartridge (Dionex, Sunnyvale, CA) and desalted with 50 mM acetic acid for 10 minutes, then separated on the capillary column (0.2X150mm Magic C18AQ 3µ 200A, Michrom Bioresources Inc, Auburn, CA) using an UltiMate™ 3000 HPLC system from LC-Packings A Dionex Co (Sunnyvale, CA). Mobile phases A and B were 0.1% formic acid in water and 0.1% formic acid in acetonitrile, respectively. Flow rate was 2 µl/min. Mobile phase B was increased from 2% to 50% in 250 min, then from 50% to 90% in 5 min, then kept at 90% for another 5 min The column was equilibrated at 2% of mobile phase B (or 98% A) for 30 min before the next sample injection. MS/MS data was acquired with a spray voltage of 2 KV and a capillary temperature of 175 °C. The scan sequence of the mass spectrometer was based on the TopTen™ method. The full scan mass resolving power was set at 30,000 to achieve high mass accuracy MS determination. The CID fragmentation energy was set to 35%. Dynamic exclusion is enabled with a repeat count of 3 within 30 s, a mass list size limit of 200, exclusion duration of 350 s and a low mass width of 0.50 and high mass width of 1.50 Da.

#### Peptide sequence analysis

The RAW data files collected on the mass spectrometer were converted to mzXML and MGF files by use of MassMatrix data conversion tools (version 1.3, http://www.massmatrix.net/download). Isotope distributions for the precursor ions of the MS/MS spectra were deconvoluted to obtain the charge states and monoisotopic m/z values of the precursor ions during the data conversion. Resulting. mgf files were searched using Mascot Daemon (version 2.3.2, Matrix Science, Boston, MA) against the Swissprot mouse database (version 57.0, 428,650 sequences; 154,416,236 residues). Trypsin was used as the enzyme and three missed cleavages were permitted. Considered variable modifications were oxidation (Met) and carbamidomethylation (Cys). The mass accuracy of the precursor ions was set to 10 ppm and the fragment mass tolerance to 0.5 Da. Accidental picking of one 13C peak was included into the search. The significance identity threshold was set at p0.05 for valid protein identification. False discovery rates (FDR) for peptide matches were estimated using the target-decoy search strategy [92,93]. All reported results are for peptides with less than 5% FDR. Proteins with a Mascot mowse score of 25 or higher containing a minimum of one unique peptide with a -b or -y ion sequence tag of five residues or better were accepted. However, identifications of proteins with one unique peptide were considered to be true positives only if: 1) the precursor ion has correct charge status and the mass accuracy is 10 ppm. 2) b and -y ion sequential tag of five or more residues were present following manual validation of MS/MS spectra.

#### Immunohistochemistry

Quadriceps were flash frozen in isopentane cooled in liquid nitrogen and 4-8 µm serial sections were cut. Tissue was fixed in 4% paraformaldehyde except for syntrophins where 1% paraformaldehyde was used. Tissue was permeabilized in 1% Triton X-100 diluted in PBS, then blocked overnight at 4^o^C in blocking solution (1% Triton X-100, 10% horse serum, in PBS). For primary antibodies raised in mouse, tissues were blocked an additional 2 hours at room temperature in blocking solution containing donkey anti-mouse Fab fragments (Jackson Immunologicals). Tissues were incubated overnight at 4^o^C in primary antibody diluted in blocking solution. Samples were then washed in PBS and incubated for 1 hour at room temperature with the appropriate secondary antibody raised in Donkey and conjugated to Cy2 or Rhodamine Red X. Some sections were incubated with alpha-bungarotoxin conjugated to Fluorescein in order to visualize NMJs. Slides were counterstained with DAPI to visualize nuclei and mounted in Vectashield. Images were captured using an Olympus BX61 microscope or a Leica TCS-NT confocal microscope.

#### Western blots

Proteins separated on 4-12% gradient SDS-PAGE gels (Invitrogen) were transferred to nitrocellulose membranes (Whatman), blocked with 5% skim milk in 0.1% Tween 20/Tris-buffered saline and incubated with primary antibodies overnight for maximum sensitivity to confirm immunodepletion of proteins. Membranes were incubated with appropriate horseradish peroxidase-conjugated secondary antibodies (Jackson ImmunoResearch) and enhanced chemiluminescence reagents (Pierce). Signal was detected on X-Ray film (RPI).

### Supporting Information

Figure S1 **Close-up view of dystroglycan staining at the membrane of muscle fibers in wild type and dystrophic quadriceps muscles.**
Micrographs from Figure 3 are reproduced after enhancement of intensity (level adjustment in Photoshop; gamma value was not changed) in the red channel in order to better visualize dystroglycan staining at the myofiber membrane (red). Squares show location of the randomly selected fiber that is enlarged at right to show the continuous dystroglycan staining at the membrane.
(TIF)

Figure S2 **DAPC proteins show unique patterns of disrupted membrane localization in *mdx* and *mdx/utr*^*-/-*^ skeletal muscle compared to wild type.** Immunolabeling of wild type, *mdx*, and *mdx/utr* ^*-/-*^ (DKO) skeletal muscle tissue sections. Arrowheads and asterisks denote non-regenerating and regenerating fibers respectively. Tissue sections were serial cut and arrowheads and asterisks correspond to the same skeletal muscle fibers shown in Figure 3A.
(TIF)

Figure S3 **Cavin-1 is reduced at the membrane of *mdx* muscle fibers.** Immunolabeling of wild type and mdx quadriceps sections for Cavin-1 (red) and nuclei (blue). Sections labeled with the secondary antibody alone (Secondary) are shown.
(TIF)

Table S1 **Calcium channel proteins identified in the β-dystroglycan immunoprecipitation from Experiment 3 by proteomics.**
(PDF)
