# Supplementary figures and images for: Identification of New Dystroglycan Complexes in Skeletal Muscle
Source: PLoS One. 2013 Aug 8;8(8):e73224. doi: 10.1371/journal.pone.0073224 (PMC3738564; doi:10.1371/journal.pone.0073224)

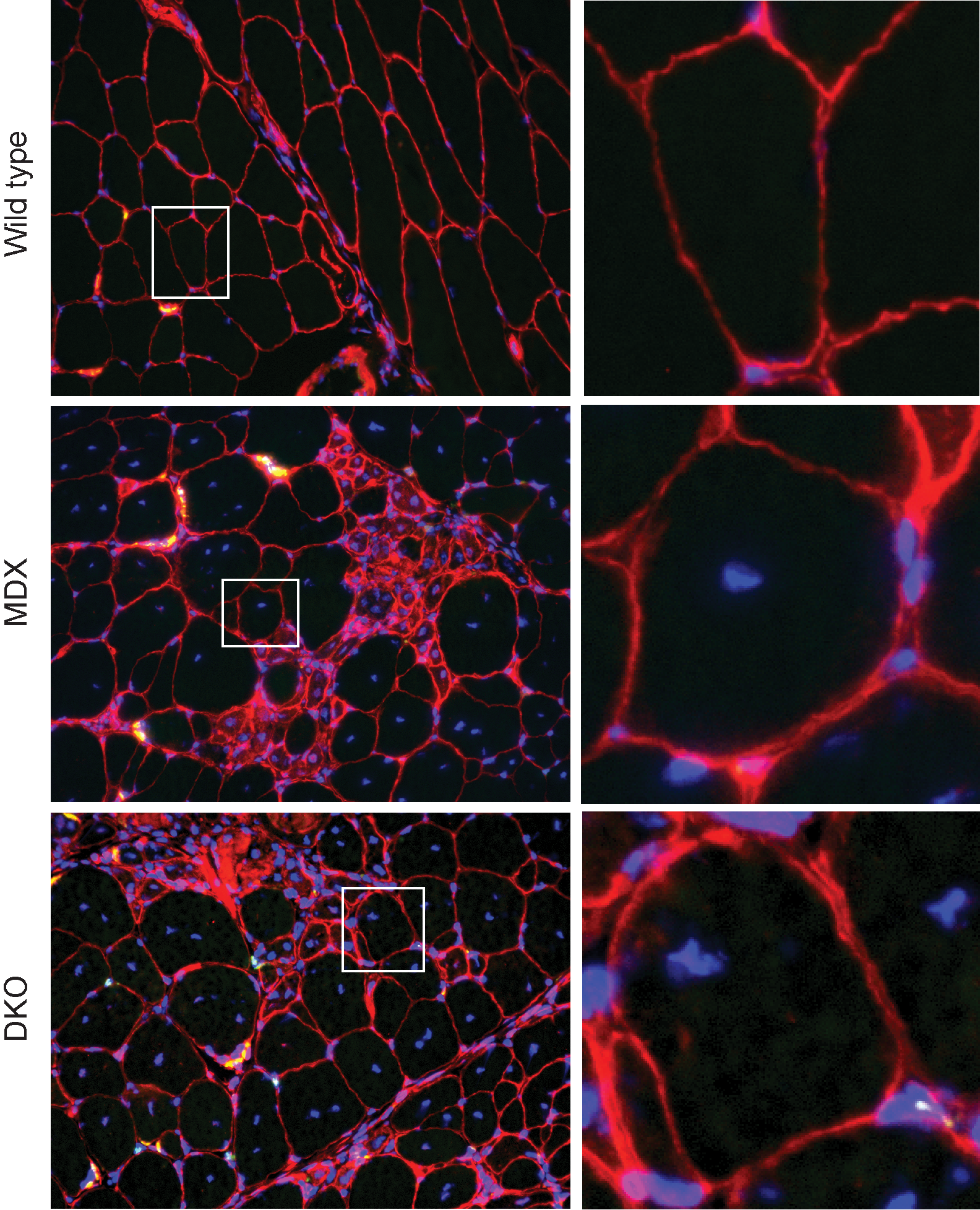

Supplement: Figure S1 — Close-up view of dystroglycan staining at the membrane of muscle fibers in wild type and dystrophic quadriceps muscles. Micrographs from Figure 3 are reproduced after enhancement of intensity (level adjustment in Photoshop; gamma value was not changed) in the red channel in order to better visualize dystroglycan staining at the myofiber membrane (red). Squares show location of the randomly selected fiber that is enlarged at right to show the continuous dystroglycan staining at the membrane. (TIF) [file pone.0073224.s001.tif]

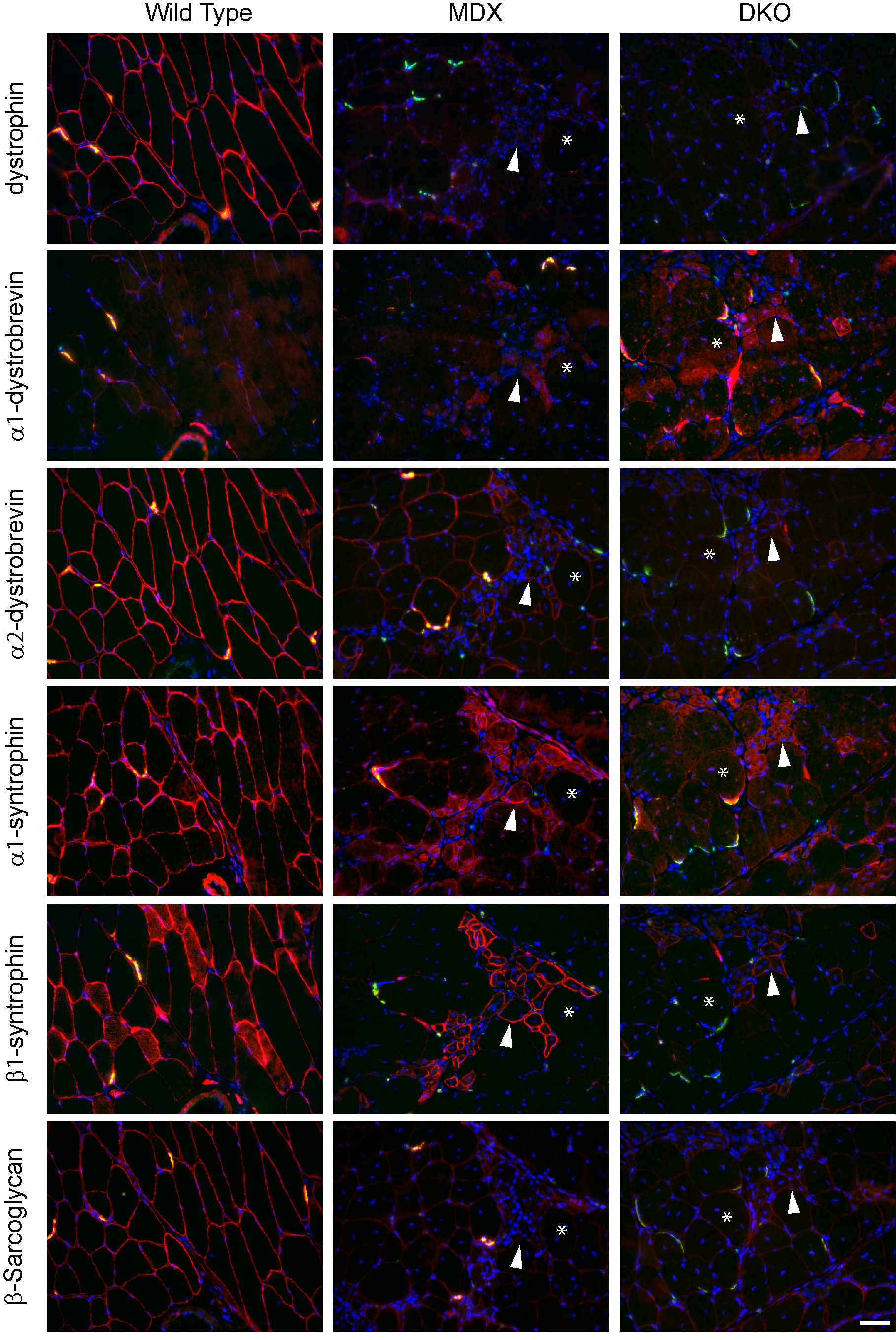

Supplement: Figure S2 — DAPC proteins show unique patterns of disrupted membrane localization in mdx and mdx/utr-/- skeletal muscle compared to wild type. Immunolabeling of wild type, mdx, and mdx/utr -/- (DKO) skeletal muscle tissue sections. Arrowheads and asterisks denote non-regenerating and regenerating fibers respectively. Tissue sections were serial cut and arrowheads and asterisks correspond to the same skeletal muscle fibers shown in Figure 3A. (TIF) [file pone.0073224.s002.tif]

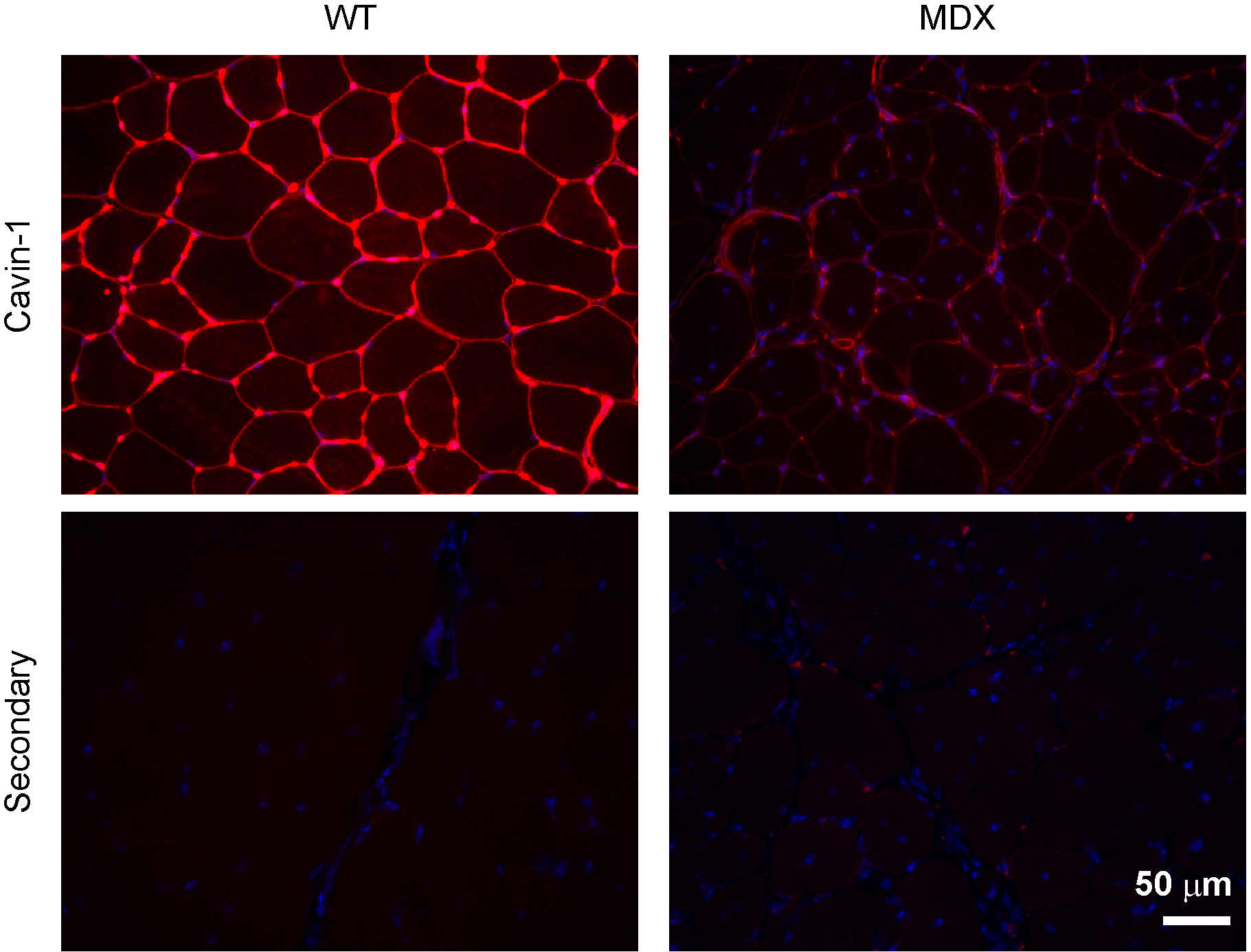

Supplement: Figure S3 — Cavin-1 is reduced at the membrane of mdx muscle fibers. Immunolabeling of wild type and mdx quadriceps sections for Cavin-1 (red) and nuclei (blue). Sections labeled with the secondary antibody alone (Secondary) are shown. (TIF) [file pone.0073224.s003.tif]
