# Supplementary material for: Identification of New Dystroglycan Complexes in Skeletal Muscle
Source: PLoS One. 2013 Aug 8;8(8):e73224. doi: 10.1371/journal.pone.0073224 (PMC3738564; doi:10.1371/journal.pone.0073224)
Supplement: Table S1 — Calcium channel proteins identified in the β-dystroglycan immunoprecipitation from Experiment 3 by proteomics. (PDF) [file pone.0073224.s004.pdf]

Table S1: Calcium channel proteins identified in the β-dystroglycan immunoprecipitation from Experiment 3 by proteomics.

SWISSPROT ID	DESCRIPTION	GENE ID	SCORE	PEP.	COV.
CAC1C_MOUSE	Voltage-dependent L-type calcium channel subunit alpha-1C	Cacna1c	50	5	1.1
CAC1E_MOUSE	Voltage-dependent R-type calcium channel subunit alpha-1E	Cacna1e	43	2	1.8
CACB3_MOUSE	Voltage-dependent L-type calcium channel subunit beta-3	Cacnb3	47	3	4.3

Score: Mascot protein confidence score; Pep: number of unique peptides identified; Cov: percent protein sequence coverage.
